# Effects of Precision Dietary Protein Reduction on Lactational Performance, Milk Fatty Acid Profile, and Antioxidant Status in Mid-Lactation Dairy Cows

**DOI:** 10.3390/ani16152431

**Published:** 2026-08-06

**Authors:** Tanakorn Damrongthai, Supreena Srisaikham, Chao Ban, Pipat Lounglawan

**Affiliations:** 1 School of Animal Technology and Innovation, Institute of Agricultural Technology, Suranaree University of Technology, Nakhon Ratchasima 30000, Thailand; 2 Faculty of Agricultural Technology, Burapha University, Sakaeo Campus, Sakaeo 27160, Thailand; supreena.sr@buu.ac.th; 3 Guizhou Testing Center for Livestock and Poultry Germplasm, Guiyang 550018, China; ban_chao@outlook.com

**Keywords:** precision feeding, superoxide dismutase, mid-lactation dairy cows, milk urea nitrogen, corn dust, monounsaturated fatty acids, nitrogen use efficiency

## Abstract

This study evaluated the effects of precision dietary crude protein (CP) reduction through corn dust substitution for soybean meal on lactational performance, milk fatty acid composition, and antioxidant status in mid-lactation dairy cows. Six Holstein Friesian cows were assigned to a replicated 3 × 3 Latin square design with three 28-day periods and fed total mixed rations containing 17.40% (TMR17), 16.29% (TMR16), or 15.18% CP (TMR15). Forage was maintained at 40% of dietary dry matter. Dry matter intake and plasma metabolites were unaffected by dietary treatment (*p* > 0.05). However, apparent digestibility of dry matter, organic matter, and crude protein declined significantly (*p* < 0.05), while acid detergent fiber digestibility showed a linear downward tendency (*p* = 0.06). Actual milk yield, lactose yield, and solids-not-fat (SNF) yield were unaffected by treatments (*p* > 0.05), but milk protein percentage decreased linearly (*p* = 0.01) as dietary CP declined. Body weight change also tended to decline (*p* = 0.09), suggestive of possible increased body reserve mobilization, although this was not confirmed with body condition score or direct metabolic indicators. Milk urea nitrogen decreased linearly (*p* = 0.05), which suggested improved nitrogen utilization. Plasma superoxide dismutase (SOD) activity, GSH-Px activity, and total antioxidant capacity were not significantly modified (*p* > 0.05); however, plasma DPPH radical scavenging capacity increased linearly with higher protein levels (*p* = 0.01; Trt: *p* = 0.02). Conversely, TMR15 improved milk lipid quality by significantly increasing monounsaturated fatty acids (*p* = 0.03; Trt: *p* = 0.05), particularly oleic acid (*p* = 0.05), whereas total saturated fatty acids remained unchanged (*p* = 0.55). Overall, reducing dietary CP improved MUFA proportions and nitrogen efficiency but decreased milk protein percentage and plasma DPPH antioxidant protection, highlighting a trade-off between production performance, animal health, and milk quality.

## Simple Summary

Feeding dairy cows excessive protein is a common commercial practice that increases feed costs and elevates environmental nitrogen pollution. This study investigated how a precision dietary strategy—gradually reducing crude protein content by replacing expensive soybean meal with corn dust (a lipid- and fiber-rich dry-milling by-product)—affects digestion, milk production, milk fatty acid profiles, and plasma antioxidant status. Six mid-lactation dairy cows were evaluated in a balanced, replicated design across three diets with decreasing protein levels (17.4%, 16.2%, and 15.2%). The results showed that lowering dietary protein and increasing corn dust inclusion linearly decreased nutrient digestibility and modestly reduced milk protein percentages, alongside a subtle decline in plasma radical scavenging capacity. However, this ingredient substitution successfully lowered milk urea nitrogen, indicating enhanced nitrogen utilization efficiency and reduced potential waste excretion. Furthermore, it significantly modified milk fat quality by reducing saturated fatty acids and expanding the proportion of heart-friendly monounsaturated fats, such as oleic acid, driven by the natural lipid matrix of the maize by-product. These findings demonstrate that while replacing soybean meal with corn dust lowers nitrogen outputs and improves milk fatty acid profiles, the concurrent reductions in digestibility and milk components represent critical economic trade-offs for dairy producers.

## 1. Introduction

In commercial dairy production, feed costs constitute the primary expense, accounting for more than 60% of total expenditures, with crude protein (CP) sources being particularly high-priced ingredients. Historically, farmers have often provided diets with protein levels exceeding standard requirements to maximize milk yield. However, this practice results in excess systemic nitrogen, which dairy cows must expend energy to excrete as milk urea nitrogen (MUN) via milk and urine, leading to both economic losses and environmental pollution [1,2]. Consequently, the concept of “precision feeding”—defined here as the formulation of diets with CP levels that closely match the actual physiological requirements of the animal rather than the application of precision technology per se—has been implemented to enhance nitrogen utilization efficiency and reduce production costs [3].

Most previous low-CP dairy cow studies have evaluated protein restriction either in isoenergetic diets balanced with rumen-protected amino acids or synthetic energy sources [4,5], while separate lines of research have examined lipid or corn by-product supplementation as an independent strategy to modify milk fatty acid profile without concurrently reducing CP [6,7]. Few studies have combined a stepwise CP reduction with a single by-product ingredient, corn dust, that simultaneously supplies both fermentable energy and preformed long-chain fatty acids. The present study therefore differs from prior work by evaluating the combined, confounded effect of replacing soybean meal with corn dust while progressively lowering CP, rather than isolating protein reduction as an independent factor. However, reducing dietary protein below standard recommendations can impair the ruminal ecosystem. Fibrolytic bacteria may experience a deficiency in ammonia nitrogen, the primary nitrogenous precursor for microbial growth and microbial protein synthesis, thereby decreasing total tract apparent digestibility of dry matter and structural carbohydrates [8,9]. This condition can induce a negative energy balance (NEB) in dairy cows, triggering body fat mobilization (lipolysis) to sustain milk production. This metabolic process releases reactive oxygen species (ROS) into the bloodstream, depleting circulating superoxide dismutase (SOD) and predisposing animals to oxidative stress and immune compromise [10,11].

To address this challenge, incorporating cost-effective energy sources such as corn dust—a by-product of corn dry-milling characterized by a lipid composition rich in oleic (C18:1n9c) and linoleic (C18:2n6c) acids—to substitute expensive protein sources like soybean meal at an optimal ratio represents a promising approach [6]. Long-chain fatty acids (LCFAs) derived from corn dust entering the small intestine may act as signaling molecules that inhibit mammary acetyl-CoA carboxylase activity, downregulating de novo fatty acid synthesis and thereby increasing the proportion of health-promoting monounsaturated fatty acids (MUFAs) in milk [12,13]. However, corn dust also increases the dietary ether extract load, which may partially coat fiber particles and further impair ADF digestibility at high inclusion levels [7]. Therefore, investigating the equilibrium between dietary protein reduction achieved through replacement of soybean meal with corn dust and the maintenance of physiological homeostasis, nutrient digestibility, and milk quality is imperative to establish sustainable dietary management guidelines for dairy cattle [14,15].

## 2. Materials and Methods

### 2.1. Ethics Statement

The experimental protocols and animal care procedures were approved by the Institutional Animal Care and Use Committee of Suranaree University of Technology (Approval No. COA. SUT-IACUC 019/2524).

### 2.2. Animals, Experimental Design, and Dietary Treatments

Six lactating Holstein Friesian dairy cows were utilized in this study. At enrollment, the cows had an average body weight (BW) of 422 ± 21.4 kg, days in milk (DIM) of 115 ± 12 d, and a milk yield of 12.3 ± 1.4 kg/d. The animals ranged from parity 1 to 2 and were free of clinical disease at the onset of the trial. The cows were allocated to two replicated 3 × 3 Latin squares (Square 1: cows A, B, and C; Square 2: cows D, E, and F). Treatment sequences were balanced to ensure that each treatment occurred once per cow and once per period within each square. Dietary treatments consisted of three total mixed rations (TMR) formulated with decreasing crude protein (CP) levels: 17% CP (TMR17), 16% CP (TMR16), and 15% CP (TMR15). Each experimental period lasted 28 days, comprising a 7-day dietary adaptation phase followed by a 21-day intensive sampling and data collection phase. Cows were housed individually in an open-sided barn subdivided into three experimental pens, providing an animal density of 6 m^2^/cow. Each pen was equipped with an individual feeding trough. Clean drinking water and commercial mineral blocks were available *ad libitum*. The concrete-floored pens were manually cleaned twice daily at 08:00 and 15:00 h. Individual body weights were recorded at the commencement and conclusion of each experimental period.

### 2.3. Feed Intake, Chemical Composition, and Apparent Digestibility

Daily feed offered and refusals were recorded to determine daily feed intake. Fresh TMR samples were collected every 5 days for DM adjustment and composite analysis. Feed samples were dried at 100 °C in a forced-air oven for 24 h (DM), or at 60 °C for 48 h, ground through a 1 mm screen, and analyzed for proximate chemical composition according to AOAC (1990) [16]. Neutral detergent fiber (NDF), acid detergent fiber (ADF), and acid detergent lignin (ADL) were determined following Goering and Van Soest [17]. Neutral detergent insoluble nitrogen (NDIN) and acid detergent insoluble nitrogen (ADIN) were determined by analyzing the nitrogen content retained in the NDF and ADF residues, respectively, following the procedure of Licitra et al. [18]. Non-fibrous carbohydrate (NFC) content, which includes starch, sugars, and soluble fiber, was calculated as: NFC (%) = 100 − (CP % + EE % + ash % + NDF %). Acid-insoluble ash (AIA) was used as an internal indigestible marker [19,20] to calculate apparent total tract digestibility coefficients.

### 2.4. Milk Production and Composition Analysis

Milk yields were recorded daily. Milk samples were collected every 5 days, pooling morning and evening samples proportionally. Aliquots were analyzed for fat, protein, lactose, solids-not-fat (SNF), total solids (TS), and MUN using an infrared milk analyzer (MilkoScan, Foss Electric, Hillerød, Denmark). The 3.5% fat-corrected milk (FCM) yield was calculated using the standard equation of Western et al. [21]: 3.5% FCM = kg milk × [0.432 + 0.162 × (fat %)]. Milk lipids were extracted and converted to fatty acid methyl esters (FAME), which were analyzed by gas chromatography (HP 6890, Hewlett Packard, Palo Alto, CA, USA) with a flame ionization detector, using helium as the carrier gas. Identification of individual fatty acids was performed by comparison with authentic FAME standards.

### 2.5. Blood Sampling and Plasma Analysis

Blood samples (10 mL) were collected from each cow via jugular venipuncture into heparinized tubes at the end of each period, prior to morning feeding. Plasma was separated by centrifugation (3000× *g*, 15 min, 25 °C) and stored at −80 °C. Plasma glucose, triglycerides, and blood urea nitrogen (BUN) were determined using commercial enzymatic colorimetric kits (Randox Laboratories, Crumlin, UK) on a semi-automated biochemistry analyzer. Plasma insulin was quantified using a bovine-specific radioimmunoassay kit (Coat-A-Count, Siemens Healthcare Diagnostics, Los Angeles, CA, USA). Superoxide dismutase (SOD) inhibition activity was determined using the WST-1 method (SOD Assay Kit-WST, Dojindo Molecular Technologies, Kumamoto, Japan) and expressed as percentage inhibition rate. DPPH (2,2-diphenyl-1-picrylhydrazyl) radical scavenging capacity was determined spectrophotometrically at 517 nm [22] and expressed as percentage scavenging activity. Glutathione peroxidase (GSH-Px) activity was measured using a kinetic colorimetric kit (Ransel, Randox Laboratories, Crumlin, UK) and expressed as U/mL. Total antioxidant capacity (TAC) was determined using the FRAP assay [23] and expressed as μmol Fe^2+^ equivalent/mL.

### 2.6. Statistical Analysis

Daily feed intake, daily milk yield, and repeated milk composition measurements collected within a period were averaged to a single period value per cow prior to statistical analysis; data were therefore analyzed as period means rather than as repeated measures over time. Data were analyzed using the MIXED procedure of SAS version 9.4 (SAS Institute Inc., Cary, NC, USA) according to a replicated 3 × 3 Latin square design. The statistical model was: Yijkl = μ + Si + C(S)j(i) + Pk + Tl + eijkl, where Yijkl is the dependent variable, μ is the overall mean, Si is the fixed effect of square (i = 1, 2), C(S)j(i) is the random effect of cow j nested within square i, Pk is the fixed effect of period (k = 1–3), Tl is the fixed effect of dietary treatment (l = 1–3), and eijkl is the residual error. The statistical model included dietary treatment and period as fixed effects, whereas cow (within square) was treated as a random effect. Least squares means (LSMeans) were compared using Tukey–Kramer adjustment for multiple comparisons. Orthogonal polynomial contrasts were used to evaluate linear and quadratic responses to decreasing dietary crude protein concentrations. Statistical significance was declared at *p* ≤ 0.05, whereas 0.05 < *p* ≤ 0.10 was considered a tendency. Given the relatively small sample size (n = 6), a post hoc power analysis was performed. The observed statistical power for the significant dry matter digestibility (DMD) response (*p* = 0.05) was estimated at 0.62, indicating that larger-scale studies are warranted to confirm the numerical trends observed in the present study.

## 3. Results

### 3.1. Dietary Ingredients and Chemical Composition

The ingredient compositions of the three experimental TMR are presented in Table 1. Soybean meal was reduced stepwise from 16.0% (TMR17) to 13.0% (TMR16) and 10.0% (TMR15), while corn dust was correspondingly increased from 12.0% to 15.0% and 18.0%. The analyzed chemical compositions and energy values are summarized in Table 2. CP content decreased progressively from 17.40% (TMR17) to 16.29% (TMR16) and 15.18% (TMR15). Ether extract, ash, NDF, ADF, and ADL increased slightly as corn dust inclusion increased, whereas NFC content fluctuated slightly between 30.28% and 30.70%. The estimated net energy for lactation (NE_Lp_) declined progressively from 1.78 (TMR17) to 1.67 (TMR16) and 1.65 Mcal/kg DM (TMR15).

### 3.2. Nutrient Intake and Apparent Total Tract Digestibility

#### 3.2.1. Dry Matter and Nutrient Intake

As summarized in Table 3, varying dietary CP levels did not significantly affect voluntary dry matter intake or most nutrient intakes (*p* > 0.05). Total DMI remained highly consistent across all groups, ranging from 14.19 to 14.54 kg DM/d (*p* = 0.75). Although crude protein intake displayed a numerical downward trend from 2529 to 2198 g/d, this decrease was statistically significant in a linear response (*p* = 0.03; Trt: *p* = 0.08) as dietary CP content declined. Similarly, fibrous fraction intakes and estimated NE_Lp_ intake (Mcal/cow) did not differ among dietary treatments (*p* > 0.05).

#### 3.2.2. Apparent Total Tract Nutrient Digestibility

Dry matter digestibility (DMD) was significantly affected by dietary CP level (*p* = 0.01), exhibiting a negative linear contrast (*p* = 0.01) as dietary protein decreased, from 66.23% in TMR17 to 64.39% in TMR16 and 62.34% in TMR15 (Table 4). Significant treatment effects and negative linear responses were also observed for organic matter (OM) (*p* = 0.04, Linear: *p* = 0.02) and CP digestibility (*p* = 0.01, Linear: *p* = 0.01), both decreasing concurrently with lower dietary protein levels. Conversely, NDF and ADF digestibility did not show overall significant treatment effects (*p* = 0.06), though NDF digestibility demonstrated a significant negative linear response (*p* = 0.02) decreasing from 65.57% to 61.53% across treatments.

### 3.3. Milk Production, Milk Composition, and Milk Urea Nitrogen

#### 3.3.1. Milk Yield and Composition

No statistically significant differences (*p* > 0.05) were observed among treatments for actual milk yield or 3.5% FCM yield (Table 5). Regarding milk composition percentages, a significant treatment effect and a negative linear response were detected for milk protein percentage (*p* = 0.01, Linear: *p* = 0.01), which decreased from 2.98% in TMR17 to 2.84% in TMR16 and 2.79% in TMR15. Other milk composition percentages and daily yields of all milk components (fat, protein, lactose, SNF, and total solids) did not differ significantly among dietary treatments (*p* > 0.05).

#### 3.3.2. Milk Urea Nitrogen

Milk urea nitrogen (MUN) concentration was significantly affected by dietary CP level (*p* = 0.05. A significant negative linear contrast (*p* = 0.03) was observed as dietary protein decreased step-wise. The highest MUN was found in TMR17 (13.91 mg/dL), followed by TMR16 (13.18 mg/dL), with the lowest in TMR15 (12.76 mg/dL).

#### 3.3.3. Body Weight and Body Weight Changes

Average body weight remained statistically comparable across groups (421 to 423 kg; *p* = 0.75). However, individual BW changes exhibited a linear tendency (*p* = 0.09). Cows on TMR17 gained +79.37 g/d, while those on lower-protein diets experienced negative BW balances (−15.87 g/d for TMR16 and −47.62 g/d for TMR15).

### 3.4. Plasma Biochemical Profiles and Antioxidative Capacity

#### 3.4.1. Blood Biochemical Indicators

Stepwise reduction in dietary CP did not significantly affect plasma biochemical indicators (Table 6; *p* > 0.05). BUN ranged from 14.1 to 14.7 mg/dL (*p* = 0.26). No significant effects were detected for plasma triglycerides, glucose, or insulin.

#### 3.4.2. Systemic Antioxidant Capacity

Plasma DPPH radical scavenging capacity exhibited a significant positive linear response (*p* = 0.01; Trt: *p* = 0.02) as dietary protein increased, with the highest value observed in the TMR17 group (12.1%) compared to TMR16 (10.5%) and TMR15 (9.7%). Conversely, plasma SOD inhibition rate, GSH-Px activity, and TAC were not significantly modified by the dietary interventions (*p* > 0.05).

### 3.5. Milk Fatty Acid Composition

#### 3.5.1. Major Fatty Acid Groups (SFA, MUFA, and PUFA)

Total saturated fatty acids (SFAs) did not differ significantly among dietary treatments (*p* > 0.05), ranging from 68.64 to 64.76 g/100 g (Table 7). Total MUFAs were significantly affected by the treatments (*p* = 0.05), exhibiting a significant positive linear contrast (*p* = 0.03) with lower values in TMR17 (29.43 g/100 g) compared to TMR16 (32.30 g/100 g) and TMR15 (33.01 g/100 g). No significant treatment effects were detected for total PUFAs (*p* = 0.26).

#### 3.5.2. Individual Distinct Fatty Acid Responses

Cis-C18:1n9 (oleic acid) showed a significant positive linear response (*p* = 0.05; Trt: *p* = 0.08), increasing from 24.52 g/100 g in TMR17 to 27.95 g/100 g in TMR15. Conversely, the proportion of trans-C18:1n9 (C18:1n9t) did not differ significantly among treatments (*p* = 0.16), although it displayed a numerical quadratic tendency (*p* = 0.09) with the lowest value observed in TMR16 (1.55 g/100 g) compared to TMR17 (1.85 g/100 g) and TMR15 (1.91 g/100 g). The proportion of C20:3n-6 exhibited a significant linear (*p* = 0.04) response, decreasing from 0.06 g/100 g (TMR17) to 0.03 g/100 g (TMR15). The proportion of conjugated linoleic acid (CLA) was not significantly affected (*p* = 0.93).

## 4. Discussion

Voluntary DMI and its relative proportion to BW did not differ significantly among the three TMR (*p* > 0.05; Table 3). This stability indicates that replacing soybean meal (16.0% to 10.0%) with corn dust (up to 18.0%) did not adversely affect dietary palatability or mastication. According to NASEM [25], the primary dietary factor limiting voluntary feed intake in ruminants is the NDF fraction. Although dietary NDF shifted slightly from 42.77% (TMR17) to 44.17% (TMR15; Table 2), this remained well below the threshold required to trigger ruminal fill and depress digesta passage rate [26].

Despite uniform DMI, apparent total tract digestibility of DM, OM, and CP declined significantly (*p* < 0.05) as dietary CP decreased, while ADF digestibility demonstrated a linear downward tendency (*p* = 0.06; Table 4). A plausible, though not directly confirmed, explanation relates to ruminal degradable protein (RDP) availability, since ruminal ammonia concentration and fibrolytic bacterial populations were not measured in this study. Soybean meal in TMR17 is highly susceptible to microbial degradation, potentially releasing ammonia nitrogen (NH3-N) that supports proliferation of cellulolytic bacteria including *Fibrobacter succinogenes* and *Ruminococcus albus* [8,9]. When cows were fed TMR15, reduced availability of degradable nitrogenous compounds may have created a subclinical nitrogen limitation within the rumen, potentially restricting fibrolytic microbial activity and contributing to the observed decline in nutrient degradation [25,26]; direct measurement of ruminal ammonia and fibrolytic bacterial activity in future studies would be needed to confirm this mechanism. Additionally, the higher ether extract content of TMR15 (4.12%; Table 2), derived from the elevated corn dust inclusion, may have partially coated fiber particles, further impairing cellulolytic enzyme access to ADF substrates [7]. This dual mechanism—nitrogen limitation and lipid coating—may partially explain the additive depression in digestibility in TMR15.

Actual milk yield and 3.5% FCM yield were statistically unaltered among treatments. It is worth noting, however, that actual milk yield declined numerically from 12.8 kg/d (TMR17) to 11.5 kg/d (TMR15), an approximate 10% reduction; given the limited statistical power afforded by the n = 6 Latin square design, this numerical decline cannot be interpreted as evidence of true biological equivalence among treatments, and a larger-scale trial would be needed to confirm whether this magnitude of change reflects a genuine production effect. A significant linear decrease was observed for milk protein percentage (*p* = 0.01), which dropped from 2.98% (TMR17) to 2.84% (TMR16) and 2.79% (TMR15). This depression in milk protein concentration directly mirrors the linear reduction in crude protein intake (2529 to 2198 g/d; Linear: *p* = 0.03; Table 3), indicating that amino acid supply to the mammary gland was likely limited in the lower-protein diets, which subsequently restricts the availability of precursors required for milk protein synthesis and transport within the mammary epithelial cells [27]. In contrast to milk protein content, the percentages and absolute daily yields of other milk components, including fat, lactose, and SNF, did not differ significantly among dietary treatments (*p* > 0.05). The linear tendency in BW change (*p* = 0.09), from +79.37 g/d (TMR17) to −47.62 g/d (TMR15), is suggestive of a possible shift toward negative energy balance (NEB) under protein restriction, consistent with reports that sub-optimal protein availability can accelerate body reserve mobilization in mid-lactation cows [28,29]; however, NEB was not directly confirmed in the present study, as body condition score, plasma non-esterified fatty acids (NEFA), and beta-hydroxybutyrate (BHBA) were not measured, and future work should include these direct indicators of fat mobilization. It should also be emphasized that dietary CP reduction in this study was confounded with several concurrent dietary changes: increasing corn dust and decreasing soybean meal inclusion, increased ether extract and fiber fractions (NDF, ADF, ADL), an altered milk and dietary fatty acid profile, and a lower estimated NE_Lp_ (Table 2). Consequently, the observed responses in milk yield, BW change, and digestibility reflect the combined effect of these concurrent changes and cannot be attributed exclusively to CP reduction per se.

MUN decreased linearly (*p* = 0.03; Trt: *p* = 0.05) from 13.91 mg/dL (TMR17) to 12.76 mg/dL (TMR15), mirroring the dietary CP gradient. This regression demonstrates that reducing dietary CP effectively lowers excess ruminal ammonia absorption, optimizing nitrogen utilization efficiency and reducing metabolic waste excretion [1,2]. All MUN values in the present study fell within the optimal range of 12–18 mg/dL [30], confirming that even the highest-protein diet (TMR17) did not induce nitrogen toxicity. The lower MUN in TMR15 implies improved synchronization between ruminal nitrogen availability and microbial requirements, an important strategy for lowering feed costs; however, confirmation of an actual reduction in environmental nitrogen loss would require direct measurement of urinary and fecal nitrogen excretion, as well as nitrogen utilization pathways in the lower digestive tract [31], which was not performed here, and this represents a priority for future work [32,33].

Regarding systemic antioxidant capacity (Table 6), plasma SOD inhibition rate, GSH-Px activity, and TAC were not significantly modified by the dietary interventions (*p* > 0.05). However, plasma DPPH radical scavenging capacity exhibited a significant positive linear response (*p* = 0.01; Trt: *p* = 0.02), peaking at 12.1 in TMR17 and decreasing step-wise to 10.5% in TMR16 and 9.7% in TMR15. The enhanced DPPH radical scavenging profile of TMR17 can be attributed to two main factors. First, soybean meal (16.0% in TMR17) is rich in legume-derived isoflavones (genistein and daidzein) with inherent radical-scavenging properties [34,35]. Second, superior nutrient synchronization in TMR17 supported positive energy balance, protecting tissues from the oxidative stress associated with lipolysis. In contrast, body reserve mobilization observed in the nutrient-restricted TMR15 group generates ROS that competitively exhaust circulating SOD reserves, suggesting reduced antioxidant defense [10,11,36]. The absence of significant effects on SOD, GSH-Px and TAC suggests that these specific enzymatic antioxidant systems may be less sensitive to the magnitude of protein variation explored here, a finding warranting investigation with longer intervention periods.

Regarding the milk lipid profile (Table 7), total SFAs were not significantly altered by dietary treatments (*p* = 0.55), remaining statistically comparable between 68.64 and 64.76 g/100 g. However, TMR15 demonstrated a clear nutritional advantage by exhibiting a significant linear increase in total MUFAs (*p* = 0.03; Trt: *p* = 0.05) from 29.43 g/100 g (TMR17) to 33.01 g/100 g (TMR15), driven by expansion of oleic acid (cis-C18:1n9) from 24.52 g/100 g to 27.95 g/100 g (*p* = 0.05). The analyzed fatty acid composition of the corn dust used in this experiment (C18:1n9c 28.7 g/100 g, C18:2n6c 52.3 g/100 g) confirms that elevated preformed LCFA supply was the primary driver of this shift. Although dietary unsaturated fatty acids undergo extensive microbial biohydrogenation in the rumen [37], the higher fat intake from 18.0% corn dust inclusion allowed a greater proportion of these dietary lipids to bypass ruminal isomerization and reach the mammary gland. Once there, circulating LCFAs may contribute to reducing de novo fatty acid synthesis, consistent with previous mechanistic studies [12,13,38]. This structural reconfiguration shifts milk toward a potentially more favorable milk fatty acid profile [39,40]. It is important to note that this shift was expressed as a proportion of total milk fat (g/100 g); because total fat yield (g/d) did not differ significantly among treatments (*p* = 0.63; Table 5), the increase in MUFA percentage does not necessarily indicate a corresponding increase in absolute daily MUFA proportion, and this distinction should be considered when interpreting the nutritional significance of the finding.

The proportion of trans-C18:1n9 (elaidic acid) did not differ significantly among treatments (*p* = 0.16), although it displayed a numerical quadratic tendency (*p* = 0.09), reaching its lowest value in TMR16 (1.55 g/100 g) and highest in TMR15 (1.91 g/100 g) (Table 7). This non-monotonic numerical pattern suggests potential minor shifts in competing biohydrogenation pathways: at intermediate corn dust levels (TMR16), the balance between dietary PUFA supply and ruminal microbial activity may have diverted more biohydrogenation flux through alternative pathways rather than elaidic acid. At the highest corn dust inclusion (TMR15), increased LCFA load may have partially altered biohydrogenation kinetics, allowing more dietary trans-18:1 isomer to flow through. From a public health perspective, the elaidic acid values observed remain low, and the concurrent increase in oleic acid represents a net health benefit for consumers [39].

The proportion of C20:3n-6 (DGLA) decreased linearly (*p* = 0.04) from 0.06 g/100 g in TMR17 to 0.03 g/100 g in TMR15. This trend aligns with reports that long-chain fatty acid supply downregulates mammary lipogenic gene expression, specifically acetyl-CoA carboxylase and stearoyl-CoA desaturase. This downregulation likely limits the elongation and desaturation cascade from C18:2n-6 toward longer-chain n-6 metabolites like DGLA [12,13,38]. Since mammary enzyme activity and gene expression were not directly quantified in the current study, this mechanism remains speculative and warrants verification through enzymatic or transcriptomic analysis in future studies. Although DGLA values were numerically small, this fatty acid has anti-inflammatory properties, and its reduction should be acknowledged as a potential nutritional trade-off of the lower-protein, higher-fat dietary strategy.

The beneficial milk fatty acid profile observed in TMR15 must be balanced against the costs to the cow: declining BW reserves, reduced nutrient digestibility, lower milk protein content, and lower DPPH-mediated antioxidant protection. From a practical standpoint, the TMR16 formulation (16.0% CP, 15.0% corn dust) may represent the most viable compromise, as it achieved MUN reduction and improved MUFA proportion without the marked BW loss or antioxidant depression observed in TMR15. This nuanced interpretation has practical implications for tropical dairy farming where both feed cost reduction and cow welfare are critical management priorities [41].

Limitations: Several limitations of the present study should be acknowledged. First, the small sample size (n = 6 cows in a 3 × 3 Latin square) constrains statistical power. Future studies should employ larger sample sizes or utilize a replicated Latin square to strengthen inference. Second, each experimental period spanned 28 days; the long-term consequences of sustained protein restriction on body condition score, reproductive performance, and immune function remain uncharacterized. Third, this study was conducted exclusively in mid-lactation Holstein Friesian cows under tropical conditions (Thailand), and results may not be directly applicable to other lactation stages, breeds, or temperate climates. Fourth, the experimental diets were not formulated to be isoenergetic or isolipid because increasing corn dust simultaneously altered dietary energy density and fatty acid composition. Consequently, responses should be interpreted as effects of the combined dietary modification rather than crude protein reduction alone. Fifth, while the fatty acid composition of the corn dust was characterized in the present study, the variability in corn dust composition between batches and sources represents a confounding factor that should be monitored in practical applications.

## 5. Conclusions

Progressively replacing soybean meal with corn dust to lower dietary CP from 17.40% to 15.18% involved a combined dietary modification that simultaneously altered CP, ether extract, fiber fractions, fatty acid profile, and estimated NE_Lp_ consequently, the observed effects on production, metabolic status, and milk quality cannot be attributed solely to CP reduction, but reflect this combined dietary change. The higher-protein TMR17 diet preserved digestibility, body weight gain, and milk protein content, and supported greater plasma antioxidant capacity, while the lower-protein TMR15 diet improved nitrogen utilization efficiency and favorably reconfigured milk fat toward cardio-protective monounsaturated fatty acids, without cost to milk yield. Taken together, these findings suggest that TMR16 (16.29% CP) offers the most practical compromise, capturing much of the nitrogen-efficiency and milk-quality benefits of protein reduction while limiting the metabolic and antioxidant trade-offs observed at the lowest protein level. Future research with larger cohorts, rumen fermentation measurements, and extended experimental periods is warranted to confirm these findings and evaluate long-term cow health and productivity outcomes.

## Figures and Tables

**Table 1:** Ingredient composition (% of DM) of the experimental total mixed rations (TMR) with different protein levels.

Ingredient (% of DM)	TMR17	TMR16	TMR15
Napier grass (*Pennisetum purpureum*)	40	40	40
Cassava chips	12	12	12
Rice bran	9	9	9
Molasses	7.5	7.5	7.5
Corn dust ^1^	12	15	18
Soybean meal	16	13	10
Urea	1.5	1.5	1.5
Mineral premix	2	2	2
Total	100	100	100

^1^ Corn dust is a dry-milling by-product generated during the mechanical processing (comprising pericarp, germ, and residual endosperm fragments removed during grain processing) of maize grain. Composition (% DM): CP 8.5%, EE 4.9%, NDF 18.3%, ADF 6.1%, ash 1.2%. Fatty acid profile: C18:2n6c 52.3%, C18:1n9c 28.7%, C16:0 13.1%, C18:0 2.8%.

**Table 2:** Chemical composition and energy values of the experimental diets (% of DM).

Item	TMR17	TMR16	TMR15
Chemical Composition			
Dry matter	92.23	92.11	92.06
CP	17.40	16.29	15.18
EE	3.88	4.09	4.12
Ash	5.51	5.66	5.83
NFC	30.44	30.28	30.70
NDF	42.77	43.68	44.17
ADF	22.31	22.99	23.28
ADL	5.11	5.61	6.19
NDIN	1.22	1.21	1.19
ADIN	0.75	0.73	0.73
TDN_1x_ (%) ^1^	68.61	67.78	66.67
DE_P_ (Mcal/kgDM) ^2^	3.23	3.06	3.03
ME_P_ (Mcal/kgDM) ^3^	2.81	2.65	2.61
NE_LP_ (Mcal/kgDM) ^4^	1.78	1.67	1.65

^1^ TDN_1X_ (%) = tdNFC + tdCP + (tdFA × 2.25) + tdNDF − 7. ^2^ DE_1X_ (Mcal/kg) = [(tdNFC/100) × 4.2] + [(tdNDF/100) × 4.2] + [(tdCP/100) × 5.6] + [(FA/100) × 9.4] − 0.3. Discount = [(TDN_1X_ − [(0.18 × TDN_1X_) − 10.3]) × Intake)]/TDN_1X_. DE_P_ (Mcal/kgDM) = DE_1X_ × Discount. ^3^ ME_p_ = [1.01 × (DE_p_) − 0.45] + [0.0046 × (EE − 3)]. ^4^ NE_Lp_ = ([0.703 × ME_p_ (Mcal/kg)] − 0.19) + ([(0.097 × ME_p_ + 0.19)/97] × [EE − 3]). Energy equations follow NRC [24].

**Table 3:** Dry matter and nutrient intake of dairy cows fed experimental diets.

Item	TMR17	TMR16	TMR15	SEM	*p* Value		
					Trt	Linear	Quadratic
DM intake (kg DM/day)	14.54	14.19	14.48	0.29	0.75	0.84	0.49
CP intake (g/day)	2529	2312	2198	82.56	0.08	0.03	0.45
NDF intake (g/day)	6222	6199	6396	145.87	0.55	0.32	0.63
ADF intake (g/day)	3243	3263	3371	74.65	0.51	0.29	0.71
NE_LP_ intake (Mcal/cow)	26.02	23.70	23.89	0.56	0.06	0.07	0.18

SEM, standard error of the mean; Trt, treatment effect; DM, dry matter; CP, crude protein; NDF, neutral detergent fiber; ADF, acid detergent fiber; NE_Lp_, net energy for lactation (predicted). No significant differences (*p* > 0.05) among treatments.

**Table 4:** Dry matter and nutrient digestibility of dairy cows fed experimental diets.

Item	TMR17	TMR16	TMR15	SEM	*p* Value		
					Trt	Linear	Quadratic
DM (%)	66.23 ^a^	64.39 ^b^	62.34 ^c^	0.56	0.01	0.01	0.97
OM (%)	72.67 ^a^	70.12 ^ab^	69.23 ^b^	0.56	0.04	0.02	0.37
CP (%)	71.27 ^a^	69.87 ^a^	66.09 ^b^	0.47	0.01	0.01	0.20
NDF (%)	65.57	63.64	61.53	0.92	0.06	0.02	0.79
ADF (%)	58.42	54.25	54.82	0.95	0.06	0.06	0.16

^a,b,c^ Means within a row with different superscripts differ significantly (*p* ≤ 0.05). SEM, standard error of the mean; Trt, treatment effect; DM, dry matter; OM, organic matter; CP, crude protein; NDF, neutral detergent fiber; ADF, acid detergent fiber.

**Table 5:** Milk production, milk composition, milk urea nitrogen, and body weight changes in dairy cows fed experimental diets with varying crude protein levels.

Item	TMR17	TMR16	TMR15	SEM	*p* Value		
					Trt	Linear	Quadratic
Milk yield (kg/day)	12.8	12.5	11.5	0.59	0.38	0.18	0.77
3.5%FCM	13.7	13.5	12.4	0.59	0.43	0.21	0.78
Fat (%)	3.96	4.01	3.98	0.11	0.63	0.62	0.45
Protein (%)	2.98 ^a^	2.84 ^b^	2.79 ^b^	0.03	0.01	0.01	0.20
Lactose (%)	4.52	4.65	4.59	0.04	0.14	0.30	0.10
SNF (%)	8.24	8.14	8.14	0.03	0.12	0.08	0.38
Total solid (%)	12.20	12.15	12.11	0.07	0.61	0.35	0.88
Fat yield (g/d)	508.16	501.25	459.36	36.93	0.63	0.38	0.75
Protein (g/d)	381.75	355.04	321.27	30.56	0.47	0.23	0.90
Lactose (g/d)	579.18	581.93	528.77	41.77	0.66	0.43	0.67
SNF (g/d)	1056.50	1019.19	937.27	74.12	0.52	0.28	0.86
Total solid (g/d)	1562.30	1521.04	1394.76	107.03	0.56	0.32	0.84
Milk urea N (mg/dL)	13.91 ^a^	13.18 ^b^	12.76 ^b^	0.25	0.05	0.03	0.60
Body weight (kg)	421	423	422	20.28	0.75	0.67	0.65
BW Change (g)	79.37	−15.87	−47.62	47.62	0.20	0.09	0.50

^a,b^ Means within a row with different superscripts differ significantly (*p* ≤ 0.05). SEM, standard error of the mean; Trt, treatment effect; BW, body weight; FCM, fat-corrected milk; SNF, solids-not-fat.

**Table 6:** Plasma biochemical indicators and antioxidative capacity of dairy cows fed experimental diets with varying crude protein level.

Item	TMR17	TMR16	TMR15	SEM	*p* Value		
					Trt	Linear	Quadratic
Biochemical indicators							
BUN mg/dL	14.7	14.4	14.1	0.21	0.26	0.13	0.74
Triglyceride, mg/dL	19.0	19.1	18.9	0.06	0.29	0.47	0.20
Glucose, mg/dL	47.7	46.5	46.2	1.22	0.78	0.54	0.87
Insulin, μIU/mL	1.5	1.5	1.5	0.06	0.84	0.72	0.68
Antioxidative capacity							
SOD, inhibition rate %	36.3	34.8	34.0	1.62	0.63	0.38	0.91
GSH-Px, U/mL	434.0	431.4	432.5	6.62	0.96	0.88	0.84
TAC, μmol/mL	7.4	7.5	7.4	0.07	0.67	0.73	0.46
DPPH scavenging, %	12.1 ^a^	10.5 ^b^	9.7 ^b^	0.25	0.02	0.01	0.30

^a,b^ Means within a row with different superscripts differ significantly (*p* ≤ 0.05). SEM, standard error of the mean; Trt, treatment effect; BUN, blood urea nitrogen; SOD, superoxide dismutase; GSH-Px, glutathione peroxidase; TAC, total antioxidant capacity; DPPH, 2,2-diphenyl-1-picrylhydrazyl.

**Table 7:** Fatty acid composition of dairy cows fed experimental diets. (g/100 g).

Item	TMR17	TMR16	TMR15	SEM	*p* Value		
					Trt	Linear	Quadratic
C4:0	2.14	2.28	2.29	0.21	0.85	0.66	0.84
C6:0	1.48	1.49	1.42	0.21	0.96	0.84	0.87
C8:0	0.90	0.82	0.80	0.23	0.94	0.79	0.93
C10:0	1.98	1.68	1.54	0.44	0.75	0.51	0.87
C11:0	0.18	0.17	0.15	0.04	0.84	0.61	0.90
C12:0	5.99	5.29	5.25	0.57	0.66	0.43	0.78
C13:0	0.15	0.13	0.12	0.03	0.78	0.55	0.87
C14:0	12.42	11.22	10.99	0.56	0.42	0.24	0.75
C14:1	0.93	0.95	0.84	0.16	0.83	0.62	0.72
C15:0	0.73	0.62	0.62	0.06	0.43	0.32	0.52
C16:0	29.89	29.18	29.32	1.63	0.96	0.84	0.84
C16:1	2.03	2.38	2.27	0.19	0.77	0.97	0.76
C18:0	12.60	12.32	12.03	0.89	0.68	0.46	0.70
C18:1n9t	1.85	1.55	1.91	0.29	0.16	0.61	0.09
C18:1n9c	24.52	27.31	27.95	1.80	0.63	0.75	0.43
C18:2n6t	0.03	0.07	0.08	0.04	0.77	0.55	0.75
C18:2n6c	1.32	1.68	1.61	0.09	0.52	0.81	0.29
C20:0	0.19	0.17	0.18	0.01	0.81	0.72	0.61
C18:3n6	0.06	0.04	0.03	0.03	0.27	0.50	0.43
CLA ^1^	0.45	0.53	0.47	0.05	0.70	0.67	0.46
C20:3n6	0.06	0.04	0.01	0.06	0.06	0.04	0.61
C22:1n9	0.10	0.11	0.04	0.04	0.59	0.88	0.15
SFA ^2^	68.64	65.37	64.76	2.77	0.55	0.32	0.79
MUFA ^3^	29.43 ^b^	32.30 ^a^	33.01 ^a^	0.97	0.05	0.03	0.38
PUFA ^4^	1.95	2.49	2.33	0.16	0.26	0.26	0.28

^a,b^ Means within a row with different superscripts differ significantly (*p* ≤ 0.05). SEM, standard error of the mean; Trt, treatment effect. ^1^ CLA = cis-9, trans-11 octadecadienoic acid. ^2^ SFA, saturated fatty acid. ^3^ MUFA, monounsaturated fatty acid. ^4^ PUFA, polyunsaturated fatty acid.

## Data Availability

The data presented in this study are available on request from the corresponding author.
